# Knocking Out *ACR2* Does Not Affect Arsenic Redox Status in *Arabidopsis thaliana*: Implications for As Detoxification and Accumulation in Plants

**DOI:** 10.1371/journal.pone.0042408

**Published:** 2012-08-06

**Authors:** Wenju Liu, Henk Schat, Mathijs Bliek, Yi Chen, Steve P. McGrath, Graham George, David E. Salt, Fang-Jie Zhao

**Affiliations:** 1 Rothamsted Research, Harpenden, Hertfordshire, United Kingdom; 2 College of Resources and Environmental Science, Hebei Agricultural University, Baoding, Hebei Province, China; 3 Department of Genetics, Faculty of Earth and Life Sciences, Vrije Universiteit, Amsterdam, The Netherlands; 4 Department of Geological Sciences, University of Saskatchewan, Saskatoon, Canada; 5 School of Biological Sciences, University of Aberdeen, Aberdeen, United Kingdom; 6 College of Resources and Environmental Sciences, Nanjing Agricultural University, Nanjing, China; Iwate University, Japan

## Abstract

Many plant species are able to reduce arsenate to arsenite efficiently, which is an important step allowing detoxification of As through either efflux of arsenite or complexation with thiol compounds. It has been suggested that this reduction is catalyzed by ACR2, a plant homologue of the yeast arsenate reductase ScACR2. Silencing of *AtACR2* was reported to result in As hyperaccumulation in the shoots of *Arabidopsis thaliana*. However, no information of the *in vivo* As speciation has been reported. Here, we investigated the effect of *AtACR2* knockout or overexpression on As speciation, arsenite efflux from roots and As accumulation in shoots. T-DNA insertion lines, overexpression lines and wild-type (WT) plants were exposed to different concentrations of arsenate for different periods, and As speciation in plants and arsenite efflux were determined using HPLC-ICP-MS. There were no significant differences in As speciation between different lines, with arsenite accounting for >90% of the total extractable As in both roots and shoots. Arsenite efflux to the external medium represented on average 77% of the arsenate taken up during 6 h exposure, but there were no significant differences between WT and mutants or overexpression lines. Accumulation of As in the shoots was also unaffected by *AtACR2* knockout or overexpression. Additionally, after exposure to arsenate, the yeast (*Saccharomyces cerevisiae*) strain with *ScACR2* deleted showed similar As speciation as the WT with arsenite-thiol complexes being the predominant species. Our results suggest the existence of multiple pathways of arsenate reduction in plants and yeast.

## Introduction

Arsenic (As) contamination affects more than 100 million people worldwide [1]. Drinking water and food are the two main routes of As exposure for humans. In South and Southeast Asia, As-contaminated groundwater has been widely used to irrigate rice crops, resulting in As toxicity, yield losses and elevated As accumulation in rice [2]–[4]. Understanding how plants take up and detoxify As is important for breeding crops with increased As tolerance or decreased As accumulation, or for developing plants for phytoremediation [5].

Arsenic in soil is present predominantly in the inorganic forms, as arsenate (As(V)) under aerobic conditions and arsenite (As(III)) under anaerobic conditions. Small quantities of organic As, such as mononmethylarsonic acid (MMA) and dimethylarsinic acid (DMA), may also be present [5]. It is well established that plant roots take up As(V) via the phosphate transporters (see review [6]). *Arabidopsis thaliana* and rice mutants defective in phosphate transport were found to accumulate much less As(V) [7], [8]. Because As(V) interferes with phosphate metabolism [9], it has to be detoxified. Reduction of arsenate is the first step of detoxification [10], [11]; the product arsenite can be extruded to the external medium [12] or complexed by thiol-rich peptides and subsequently stored in the vacuoles [10], [13]–[18]. Free As(III) is highly toxic as it reacts with vicinal dithiols of proteins altering their structure or catalytic functions [9], [19]. Cytosolic free As(III) must be maintained at a low level to avoid toxicity; this is achieved by complexation with phytochelatins (PCs) in most plant species [13]–[15], [20], [21]. The As(III)-PC complexes are transported into the vacuoles by ABCC transporters (Song et al., 2010). An exception to the thiol-dependent As(III) detoxification is the As hyperaccumulator *Pteris vittata*, which is able to store uncomplexed As(III) in the vacuoles [22]–[26].

Many plant species have a high capacity to reduce As(V) [6]. However, how plants reduce As(V) to As(III) remains unclear. It is thought that non-enzymatic reduction of As(V), e.g. by reaction with reduced glutathione (GSH), would be too slow to account for the rate of reduction observed in the plant extracts [18]. The plant homologues of the yeast arsenate reductase (Acr2p) have been cloned from *Arabidopsis thaliana*, *Holcus lanatus*, rice (*Oryza sativa*) and *Pteris vittata* [18], [26]–[28]. ACR2 belongs to the protein tyrosine phosphatases, which are involved in cell division and cycle (CDC) [29], [30]. In fact the *Arabidopsis* ACR2 homologue was initially identified as a dual-specificity CDC25 phosphatase [31]; the ability of this enzyme to reduce As(V) may be an adventitious property [30]. However, the phosphatase activity was found in AtACR2 and OsACR2, but not in ScACR2 or PvACR2 [26], [27]. Plant ACR2s are capable of reducing As(V) when the genes are expressed heterologously in *E. coli* [26], [27]. *In vitro* assays also showed an apparent decrease in the arsenate reduction activity from the crude extract of the *A. thaliana* T-DNA insertion lines of *AtACR2* (*AtCDC25*) compared with wild-type [18], [32]. These insertion mutants show no abnormal phenotypes when grown under the As-free control conditions [32], but are more sensitive to hydroxyurea, a substance that stalls the replication of DNA [33]. When challenged with As(V), the mutants show subtle phenotypes with slightly increased sensitivity at low As(V) concentrations [18]. Dhankher et al. [28] reported that silencing *AtACR2* using RNAi resulted in greatly increased sensitivity to As(V) and markedly increased As accumulation in the shoots of *A. thaliana*. They proposed that the apparent “As hyperaccumulation” resulted from knocking down of arsenate reduction in the roots, so that As(V) could be efficiently loaded into the xylem vessels via the phosphate transporters, unlike the wild-type plants in which As is sequestered in the root vacuoles as As(III)-thiol complexes. It should be noted that Bleekers et al. [18] observed the opposite with regard to As translocation from roots to shoots in the T-DNA insertion mutants, although the difference with wild-type was relatively small. Apart from this discrepancy, none of the above-mentioned studies has investigated the *in vivo* As speciation in the *acr2* mutant plants; this information is necessary to determine the *in planta* function of ACR2 with regard to arsenate reduction, especially because this is a key step of As detoxification.

In the present study, we determined As accumulation and speciation in *A. thaliana Atacr2* mutants, *AtACR2* overexpressing lines and wild-type plants after exposure to As(V). For comparison, As speciation was also analyzed in the yeast strain with *ScACR2* being deleted. Our results show no significant effect of knocking out *ACR2* on the arsenic redox status in *A. thaliana* and yeast, suggesting the existence of other arsenate reduction enzymes.

## Results

### Expression of *AtACR2*

**Figure 1:**
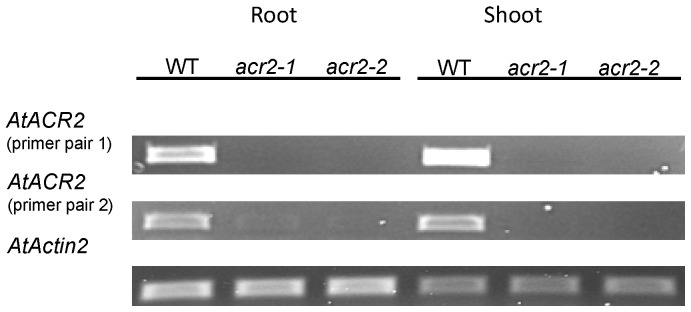
RT-PCR of *AtACR2* in wild-type *Arabidopsis thaliana* and T-DNA insertion lines of *AtACR2* gene.

RT-PCR shows that *AtACR2* was expressed in both roots and shoots of WT plants, but not in the two T-DNA insertion lines (Figure 1).

### Time-course of Arsenate Reduction

**Figure 2:**
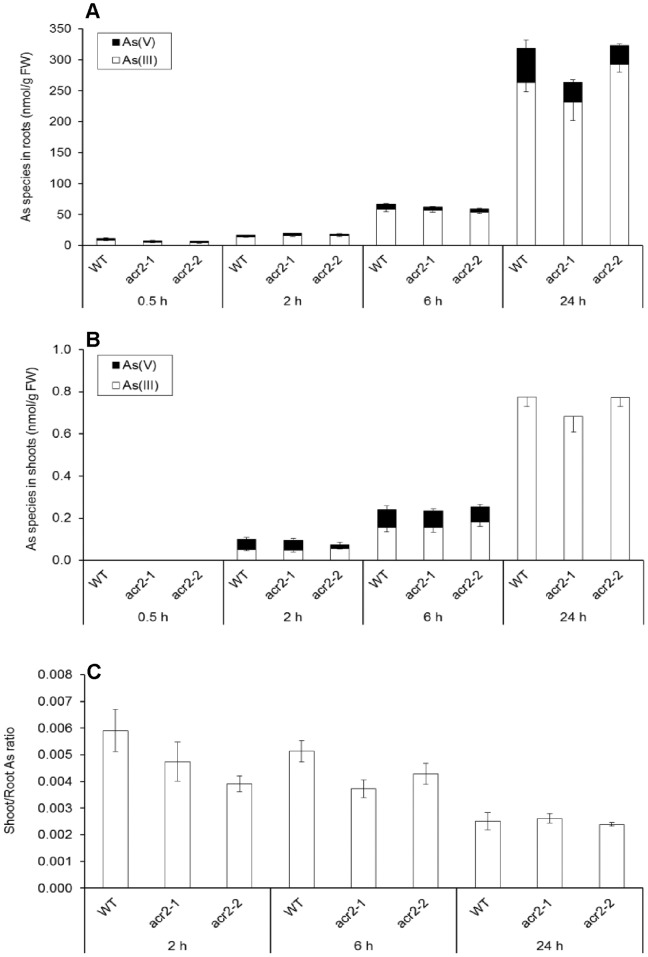
Time-course of arsenate (As(V)) reduction in *Arabidopsis thaliana* wild-type and *AtACR2* mutants. As speciation in roots (A), shoots (B) and the ratio of shoot to root As concentration (C). Plants were exposed to 5 µM As(V) for 0.5–24 h.

Because the expression of *ACR2* was shown to be induced to certain extent by arsenate exposure [18], [27], we first tested *in planta* As speciation in a time-course experiment. WT and *AtACR2* knockout mutants (*acr2-1* and *acr2-2*) were exposed to a relatively low concentration (5 µM) of arsenate for 0.5–24 h. Arsenic accumulation increased with the exposure time; at 0.5 h As was not detectable in the shoots (Figure 2A, B). Arsenate was rapidly reduced to arsenite (As(III)), with the percentage of As(III) in the roots increasing from 68% at 0.5 h to 90% at both 6 and 24 h, and in the shoots from 62% at 2 h to 100% at 24 h. Overall there was no significant difference between WT and the two *acr2* lines in either the total accumulation of As or the percentage of As(III). The majority of As was retained in the roots, resulting in a small ratio of shoot to root As concentrations (Figure 2C). This ratio decreased with the exposure time, but there was no consistent difference between WT and the mutants. In a further experiment, plants were exposed to 25 µM arsenate (a medium level) for 1 week (in the presence of 0.4 mM phosphate). Again, there was no significant difference between WT and the mutants in As accumulation, distribution or speciation, with As(III) being the predominant form in both roots (94%) and shoots (100%) (Figure S1).

### Response to Different Doses of Arsenate Exposure

**Figure 3:**
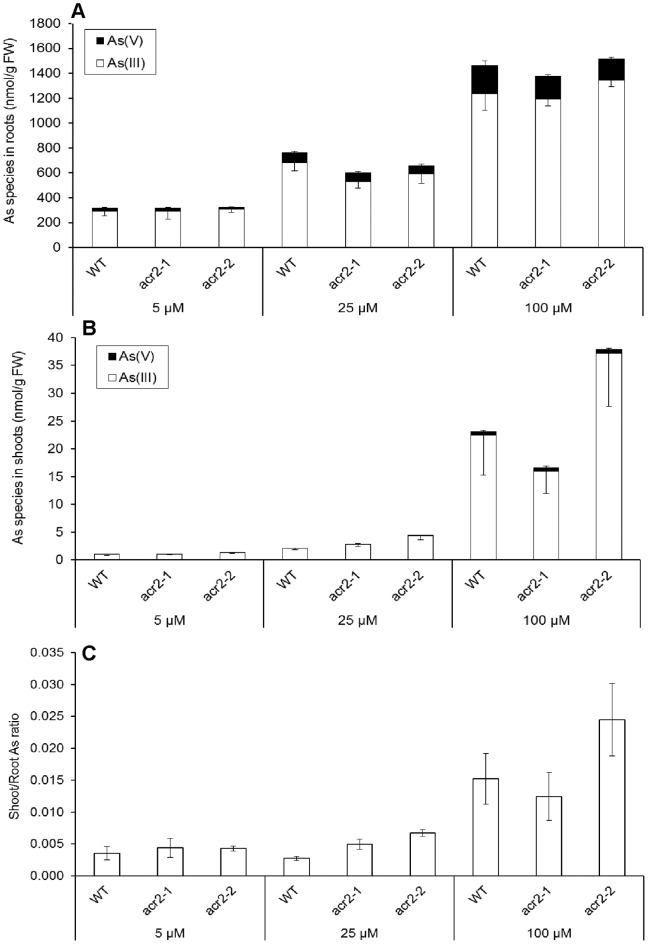
Dose-response of arsenate (As(V)) reduction in *Arabidopsis thaliana* wild-type and *AtACR2* mutants. As speciation in roots (A), shoots (B) and the ratio of shoot to root As concentration (C). Plants were exposed to 5–100 µM As(V) for 24 h.

To investigate if arsenate reduction is affected by the dose of arsenate exposure, three concentrations of arsenate were used, representing low (5 µM), medium (25 µM) and high (100 µM) doses of arsenate exposure [18]. After exposure for 24 h, most of the As accumulated in the roots and shoots was in the form of As(III) (Figure 3A, B). The percentage of As(III) in the roots decreased slightly from 94% to 86% with increasing concentration of arsenate exposure, whereas the percentage in the shoots increased slightly from 92% at 5 µM arsenate to 98% at both the 25 and 100 µM arsenate treatments. Regardless of the level of arsenate exposure, the As(III) percentage was similar between WT and the *acr2* mutants. The ratio of shoot to root As concentrations was also similar between WT and the mutants (Figure 3C). Larger ratios were obtained at 100 µM arsenate than the other two arsenate treatments.

### Arsenite Efflux

**Figure 4:**
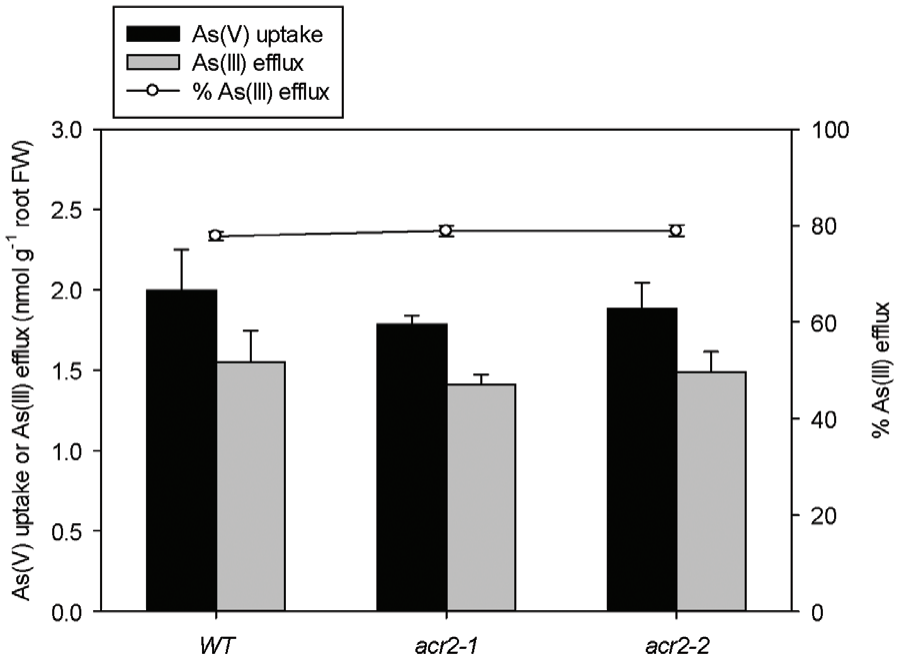
Arsenate (As(V)) uptake and arsenite (As(III)) efflux in wild-type and *AtACR2* mutants of *Arabidopsis thaliana*. Plants were exposed to 5 µM As(V) in a phosphate-free nutrient solution for 6 h.

Previous studies [12], [14] have shown that plant roots extrude As(III) to the external solution following uptake of As(V). If As(V) reduction is impaired in the *acr2* mutants, it may be expected that As(III) efflux will also decrease. As(V) uptake and As(III) efflux were quantified by monitoring the changes in As speciation in the nutrient solution after 6 h exposure to As(V) at an initial concentration of 5 µM (without phosphate). The exposure time was chosen as it was in the middle of the linear uptake phase (see Figure 2). Both As(V) uptake and As(III) efflux were similar between WT and the *acr2* mutants with As(III) efflux accounting for 77–79% of the As(V) uptake in all three lines (Figure 4).

### Arsenic Speciation in AtACR2 Overexpression Lines

**Figure 5:**
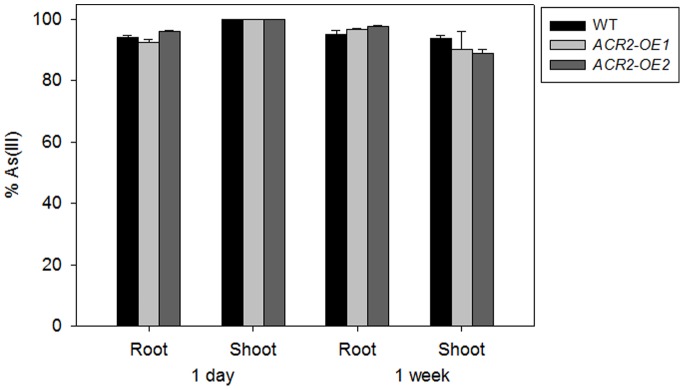
Effect of *AtACR2* overexpression on the percentage of As(III) in *Arabidopsis thaliana*. Plants were exposed to 5 µM As(V) for 1 day or 1 week.

Arsenic speciation was analyzed in WT and two lines of *A. thaliana* overexpressing *AtACR2* after exposure to 5 µM As(V) for 1 day or 1 week. Most of the As(V) taken up was reduced to As(III), representing 92–98% in roots and 89–100% in shoots (Figure 5). However, there was no significant increase in the As(III)% in the two *ACR2* overexpressing lines compared with WT.

### Arsenic Tolerance

**Figure 6:**
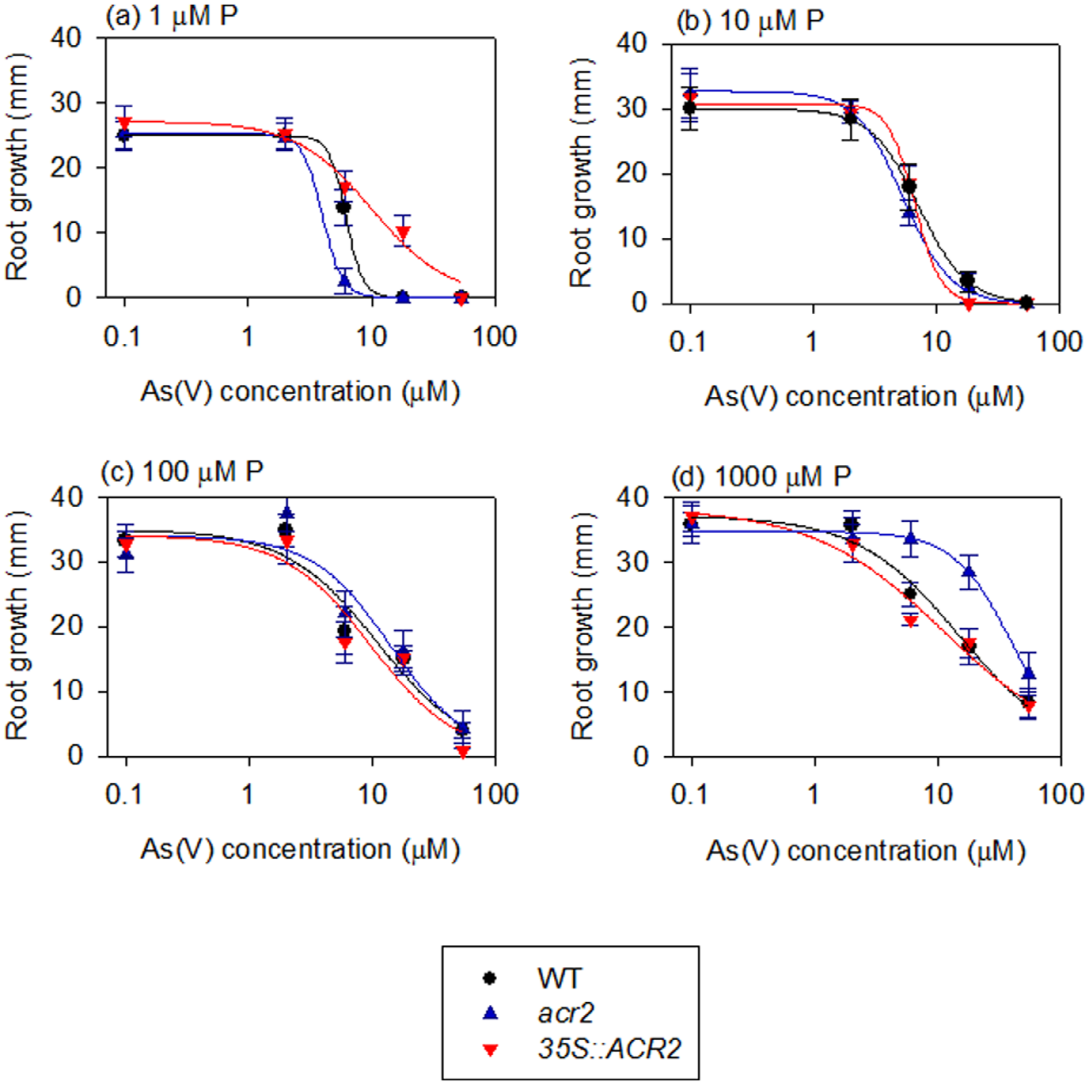
Root elongation as affected by arsenate and phosphate concentrations in wild-type, *AtACR2* knockout mutant (*acr2-1*) and overexpression line (*ACR2-OE1*).

Arsenic tolerance was tested in WT, *acr2-1* and *ACR2-OE1* lines using a root elongation assay. Significant differences between WT and the knockout mutant or the overexpression line were observed at either a very low (1 µM) or a very high (1000 µM) phosphate concentration, but not at medium phosphate concentrations (10 and 100 µM) (Figure 6). At 1 µM phosphate, the knockout mutant was more sensitive to As than WT, whereas the overexpressing line was more tolerant to As than WT. At 1000 µM phosphate, the knockout mutant became more tolerant to As than WT, whereas the overexpressing line had a level of tolerance similar to that of WT. In contrast to root growth, no clear difference in shoot growth was observed.

### Arsenic Speciation in Yeast

**Figure 7:**
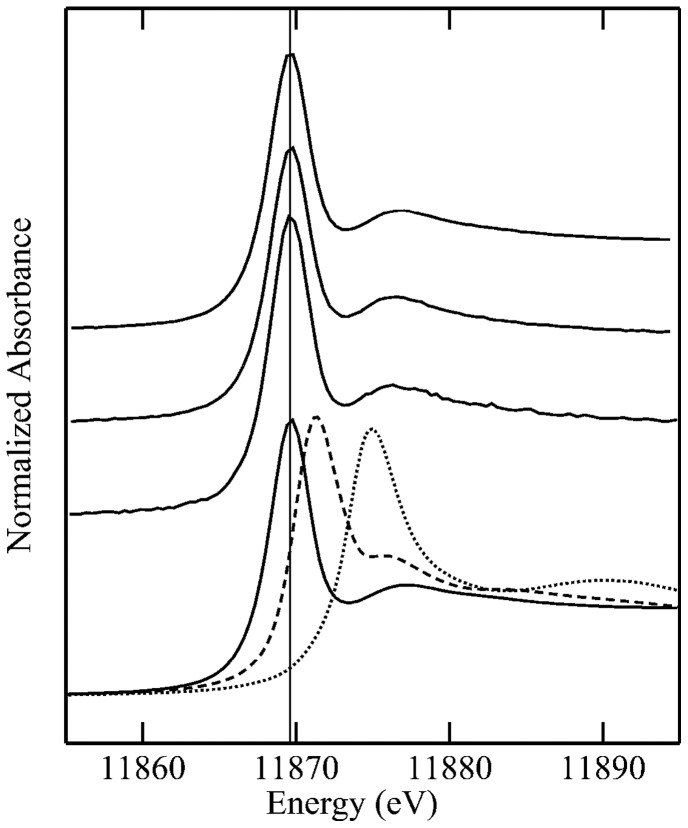
Arsenic speciation in yeast by X-ray absorption near edge structure (XANES). From top to bottom: yeast wild-type, *ScACR2* deletion strain (RM1), RM1 expressing *PvACR2* and standard compounds (solid line, As(GS)_3_ at pH 7; dashed line, arsenite pH7; dotted line, arsenate at pH 7). Fitting with linear combination of standard spectra gave essentially a single component of As(GS)_3_ indicating that all three samples contained As(III) coordinated by three aliphatic thiolate ligands.

Three strains of yeast, WT, Δ*ScACR2* in which the *ACR2* gene was deleted and +*PvACR2* (Δ*ScACR2* expressing the *Pteris vittata ACR2* gene), were analyzed for As speciation using X-ray absorption near edge structure (XANES) after exposure to As(V). The three strains showed very similar XANES spectra with the absorption edge coinciding with that of the As(III)-triglutathione complex and clearly different from the As(V) peak (Figure 7).

## Discussion

In the present study, we show that the ability to reduce arsenate has not been compromised in the *A. thaliana acr2* mutants. Both the mutants and WT plants were able to reduce arsenate rapidly and to a similarly large extent. This was confirmed by both the time-course and the arsenate dose experiments. Conversely, ectopic overexpression of *AtACR2* did not enhance arsenate reduction either. Heterologous expression of the *E. coli* arsenate reductase ArsC in *A. thaliana* also did not significantly change the As redox status in the plant [11]. Similarly, the yeast *acr2* deletion mutant was also able to reduce arsenate to the extent observed in the WT strain. These results can be explained if there exist other enzymes for arsenate reduction. Alternatively, arsenate reduction may be carried out via non-enzymatic reactions (e.g. by reactions with reduced GSH), although kinetically such reactions are likely to be too slow to account for the rapid reduction occurring in plants [18], [34]. Either way, the role of AtACR2 in arsenate reduction appears to be redundant.

Not only was there no significant difference in As speciation between *AtACR2* knockout mutants and the WT plants (which was not investigated by Dhankher et al. [28]), we also observed no difference between the mutants and WT in total As accumulation in leaves or translocation from roots to shoots over the 24 hr or 1 week of As exposure in our experiments. This contrasts with the large increase in shoot As observed in *A. thaliana* lines knocked down for *ACR2* using RNAi as reported by Dhankher et al. [28]. Such differences might be due to the different experimental conditions used. Here, we report results for As accumulation in leaves of 4-week old plants grown hydroponically in Hoagland’s solution after 24 hr or 1 week exposure to As. Whereas, Dhankher et al. [28] report As concentrations in shoots of plants grown in Petri dishes for 3-weeks on agar solidified ½ MS medium (containing sucrose) in the presence of 100 µM As. Further, the methods used to reduce expression of *ACR2* in our study and that of Dhankher et al. [28] are different. In the study reported here *ACR2* loss-of-function alleles are generated by the selection of lines with T-DNA insertions in *ACR2* that produce genetically stable and specific loss-of-function *acr2* mutants which have very limited risk of interference with non-target genes. In contrast, Dhankher et al. [28] utilized RNA interference (RNAi) to knock down expression of *ACR2*. Because the mechanism of RNAi is based on sequence homology between the target gene and the RNAi construct this approach carries an increased risk of interference with expression of non-target genes, though Dhankher et al. [28] reduced this risk by using the more specific 3′UTR of *ACR2* to generate the RNAi construct. For a review of these different approaches to the generation of reduced function alleles of genes of interest see [35].

It is possible that there are no specific arsenate reductases; rather, reduction of arsenate is carried out adventitiously by enzymes having other essential functions, or even non-enzymatically. Several mammalian enzymes appear to be capable of reducing arsenate *in vitro* in the presence of an appropriate thiol compound, including purine nucleoside phosphorylase (PNPase), glyceraldehyde-3-phosphate dehydrogenase (GAPDH) and glycogen phosphorylase (GPase) [36]–[38]. More recent studies by these authors show that the phosphorolytic enzymes, including those mentioned above, do not reduce arsenate *per se,* but rather convert As(V) into arsenylated products (i.e. ribose-1-arsenate complexes), which are unstable and more readily reducible by thiols than inorganic As(V) [39], [40]. Similarly, mitochondrial ATP synthase can catalyze the formation of ADP-As(V), which is subsequently reduced to As(III) by mono-or di-thiols [41]. This explains the ability of isolated mitochondria to reduce As(V) in the absence of ACR2/CDC25 enzymes which are localized in the cytosol. It is probable that plant phosphorolytic enzymes can also facilitate As(V) reduction in the same way, although this remains to be tested.

Additionally, Rathinasabapathi et al. [42] reported that a cytosolic triosephosphateisomerase (TPI) isolated from *P. vittata* may be involved in arsenate reduction directly or indirectly. Expression of the *PvTPI* gene in the *E. coli* strain lacking ArsC increased its arsenate resistance and the percentage of arsenite in the cellular arsenic content. It is possible that TPI may facilitate As(V) reduction with a similar mechanism as the phosphorolytic enzymes described above.

Given that the function of AtACR2 in As(V) reduction appears to be redundant, how can the altered As sensitivity in the knockout mutants be explained? The yeast *ScACR2* deletion mutant also shows increased arsenate sensitivity [26], [29]. One possible explanation may lie in its cellular localization, where a localized As(V) reduction may protect certain metabolic processes. It is also tempting to speculate that ACR2 may promote the formation of As(III)-thiol complexes, resulting in enhanced As tolerance. This speculation is based on the results of Bleeker et al. [18], who measured the *in vitro* ACR2 activity by quantifying the formation of As(III)-GS_3_ and obtained a decreased activity in the *AtACR2* knockout mutants. It could also explain the much higher As-induced PC synthesis rate in the As-tolerant ecotype of *Holcus lanatus* [18]. Furthermore, this interpretation may help explain the surprising results of an increased Cd tolerance in *A. thaliana* expressing the *E. coli* arsenate reductase gene *ArsC* [43], given that Cd, a non-redox active metal, also requires complexation with thiols for detoxification [44]. However, an ACR2-enhanced As(III)-thiol complexation does not appear to occur in yeast, as the XANES spectra showed the dominance of As(III)-GS_3_ in both the yeast WT and the *acr2* deletion mutant (Figure 7). In yeast arsenic resistance requires both efflux of As(III) from the cell by ACR3 and transport of As(III)-GS_3_ into the vacuole by YCF1 [45], [46]. In yeast lacking a functional ACR2 it is clear from our results that As(V) is still efficiently reduced to As(III) but that this As(III) is unable to be effluxed from the cell since As hyperaccumulates in the *acr2* yeast mutant [26]. It is possible that the As(III)-GS_3_ that over accumulates in *acr2* may be compartmentalized to some extent in the vacuole via the action of YCF1, but further studies would be needed to confirm this.

In conclusion, knocking out *AtACR2* does not affect arsenate reduction, nor As accumulation and distribution in *A. thaliana*. The results suggest the existence of other enzymes capable of arsenate reduction or of facilitating arsenate reduction in *A. thaliana* and yeast. Manipulating *ACR2* in plants is therefore unlikely to induce As hyperaccumulation for the purpose of As phytoremediation.

## Materials and Methods

### Plant Culture and Experiments Set-up

The *Arabidopsis thaliana* lines used in the present study were wild-type (WT, Col-0), two homozygous T-DNA lines with insertions in the *AtACR2* gene (At5G03455.1) (SALK_143282 and GABI-Kat 772G06, named *acr2-*1and *acr2-2*, respectively, in the present study), and two independent *AtACR2* overexpression lines driven by the 35S promoter. Both lines have been described previously [18]. Seeds were germinated in 0.5 ml Eppendorf tubes filled with 1.5% agar medium; the tubes were cut at about 0.8 cm from the bottom and inserted into holes in the covers of 600 ml plastic boxes. The boxes were filled with a modified 1/10 strength Hoagland nutrient solution containing 0.6 mM KNO_3_, 0.4 mM (NH_4_)_2_HPO_4_, 0.1 mM MgSO_4_, 0.4 mM Ca(NO_3_)_2_, 2 µM H_3_BO_3_, 0.06 µM CuSO_4_, 0.36 µM MnCl_2_, 0.1 µM ZnSO_4_, 0.04 µM NaMoO_4_, 20 µM FeNaEDTA. The pH of the nutrient solution was buffered at 5.5 with 2 mM MES (2-morpholino-ethanesulphonic acid, pH adjusted with KOH). Nutrient solution was renewed every 3 d after germination. All the experiments were performed in a growth chamber (20/20°C day/night temperature, light intensity 120 µmol m^−2 ^S^−1^, 16 h photon period per day, relative humidity 70%). After pre-culture for 4 weeks, 4 seedlings of WT and the T-DNA insertion lines were collected to check the expression of *AtACR2*. Other seedlings were used in the experiments described below.

### Time-course of Arsenate Exposure

WT and the two knockout mutants (*acr2-1* and *acr2-2*) were exposed to 5 µM arsenate in the background of the nutrient solution described above, except that the phosphate concentration was lowered to 25 µM. At 0.5, 2, 6 and 24 h, plants were harvested. Each time point was replicated four fold. Roots were desorbed of the apoplastic As in an ice-cold solution containing 1 mM K_2_HPO_4_, 0.5 mM Ca(NO_3_)_2_ and 5 mM MES (pH 5.5) for 10 mins. Roots and shoots were separated, weighed and frozen in liquid N_2_ before analysis of As speciation.

### Exposure to Different Arsenate Concentrations

WT and the two knockout mutants (*acr2-1* and *acr2-2*) were exposed to 5, 25 and 100 µM arsenate for 24 h in the nutrient solution containing 25 µM phosphate. Each arsenate treatment was replicated in four pots. Plants were sampled as described above.

### Arsenite Efflux

WT and two knockout mutants (*acr2-1* and *acr2-2*) were exposed to 5 µM arsenate in the nutrient solution without phosphate for 6 h. Each treatment was replicated in four pots. Aliquots of 0.5 ml uptake solution were removed from each pot at 6 h, diluted with 4.5 ml phosphate buffer solution (PBS) containing 2 mM NaH_2_PO_4_ and 0.2 mM Na_2_-EDTA (pH 5.5) and filtered through a 0.45 µm membrane filter before being used for As speciation analysis. At the end of the experiment, the volume of the uptake solution and the fresh weight of roots were recorded.

### Arsenic Speciation in *AtACR2* Overexpression Lines

Seedlings of WT and two lines of *A. thaliana* overexpressing *AtACR2* (named *ACR2-OE1* and *ACR2-OE2*) were exposed to 5 µM arsenate in the nutrient solution free of phosphate for 1 day or 1 week. Each line was replicated in four pots. Plants were harvested for As speciation as described above.

### Expression of *AtACR2*

Total RNA were isolated with TRIzol Reagent (GIBCOBRL) and cDNA was synthesized by SuperScript™ III Reverse Transcriptase (Invitrogen) according to manufacturer’s instructions. RT-PCR was done with the programme of 95°C 5 min, 95°C 30 sec, 58°C 30 sec, 72°C 60 sec, 30 cycles, 72°C 10 min, 4°C stop. Two pairs of primers were used in the PCR for *AtACR2*, primer pair one: forward 5′-ACATCACCTCTACTCAGCTT-3′ and reverse 5′-AGGTCCCAATATGGTTTAGT-3′; primer pair two: forward 5′-AGGTTCGTGGCCCTACTTGT-3′ and reverse 5′-AAGTTTGGTGTTTGTGCTCC-3′. The expression of actin was used as a control (primer, forward 5′-TCACAGCACTTGCACCAAGCA-3′, reverse 5′-AACGATTCCTGGACCTGCCTCA-3′).

### Analysis of Arsenic Speciation

Shoots and roots were ground in liquid nitrogen to fine powder in a mortar and pestle. The finely ground materials (∼0.1 g) were extracted with 10 ml phosphate buffer solution (2 mM NaH_2_PO_4_ and 0.2 mM Na_2_-EDTA, pH 5.5) for 1 h under sonication in a 4°C cold room. The extract was filtered firstly through No.42 Whatman filter paper and then through a 0.2 µm membrane filter. Arsenic speciation was determined using HPLC-ICP-MS (Agilent LC1100 series and Agilent ICP-MS 7500ce, Agilent Technologies, Santa Clara, CA, US). Arsenic species (arsenite, arsenate, DMA and MMA) were separated by an anion-exchange column (Hamilton PRP X-100, fitted with a guard column; Reno, NV, USA) with a mobile phase of 4.4 mM NH_4_H_2_PO_4_, 4.4 mM NH_4_NO_3_ and 0.2 mM Na_2_-EDTA (pH 6.2), run isocratically at 0.7 ml min^−1^. The solution from the separation column was mixed continuously with an internal standard solution (germanium) before being introduced to a concentric nebulizer and a water-jacketed cyclonic spray chamber of the ICP-MS. Signals at *m/z* 75 (As) were collected with a dwell time of 300 ms, and of 35 (Cl) and 72 (Ge) with a dwell time of 100 ms. Possible polyatomic interference of ArCl on *m/z* 75 was removed by the Agilent Octopole Reaction System operating in the helium gas mode. Arsenic species in the samples were quantified by external calibration curves with peak areas after normalization with the counts of the internal standard Ge. Arsenic speciation in the uptake solution was determined in the same way. The extraction and analytical procedure did not change the As speciation in the sample, as confirmed by the results from spiking of arsenate or arsenite to As-free plant samples. No methylated As species (DMA or MMA) were detected in any samples.

### Arsenic Speciation in Yeast

Yeast cultures were grown and exposed to 0.5 mM arsenate as described previously by Ellis et al. [26]. This concentration did not cause toxicity to the yeast strains used in the present study [26]. Freshly cultured yeast samples were transported to the Stanford Synchrotron Radiation Lightsource (SSRL) for XAS analysis of bulk samples. Data were collected on SSRL beamline 9-3 which is equipped with Si(220) monochromator crystals. Harmonic rejection was accomplished by setting the energy cutoff of the upstream specular beamline optics to 15 keV. XAS data were measured as the fluorescence excitation spectrum by monitoring the As Kα intensity using a 30-element germanium array detector (Canberra Industries, Meriden, CT) and the sample was maintained at ∼10 K in a CF1204 liquid helium flow cryostat (Oxford Instruments, Concord, MA). Incident and transmitted X-ray intensity was monitored using N_2_-filled gas ionization chambers. XAS data analysis followed our previously established procedures [10].

### Statistical Analysis

The significance of the difference between lines was determined by analysis of variance (ANOVA). Where necessary, data were transformed logarithmically to obtain homogeneity of variances.

## Supporting Information

Figure S1 **Arsenate (As(V)) reduction in** ***Arabidopsis thaliana*** **wild-type and** ***AtACR2*** **mutants.** As speciation in roots (A), shoots (B) and the ratio of shoot to root As concentration (C). Plants were exposed to 25 µM As(V) in nutrient solution with 0.4 mM phosphate for 1 week.
(PDF)
