# Supplementary material for: Knocking Out ACR2 Does Not Affect Arsenic Redox Status in Arabidopsis thaliana: Implications for As Detoxification and Accumulation in Plants
Source: PLoS One. 2012 Aug 6;7(8):e42408. doi: 10.1371/journal.pone.0042408 (PMC3412857; doi:10.1371/journal.pone.0042408)
Supplement: Figure S1 — Arsenate (As(V)) reduction in Arabidopsis thaliana wild-type and AtACR2 mutants. As speciation in roots (A), shoots (B) and the ratio of shoot to root As concentration (C). Plants were exposed to 25 µM As(V) in nutrient solution with 0.4 mM phosphate for 1 week. (PDF) [file pone.0042408.s001.pdf]

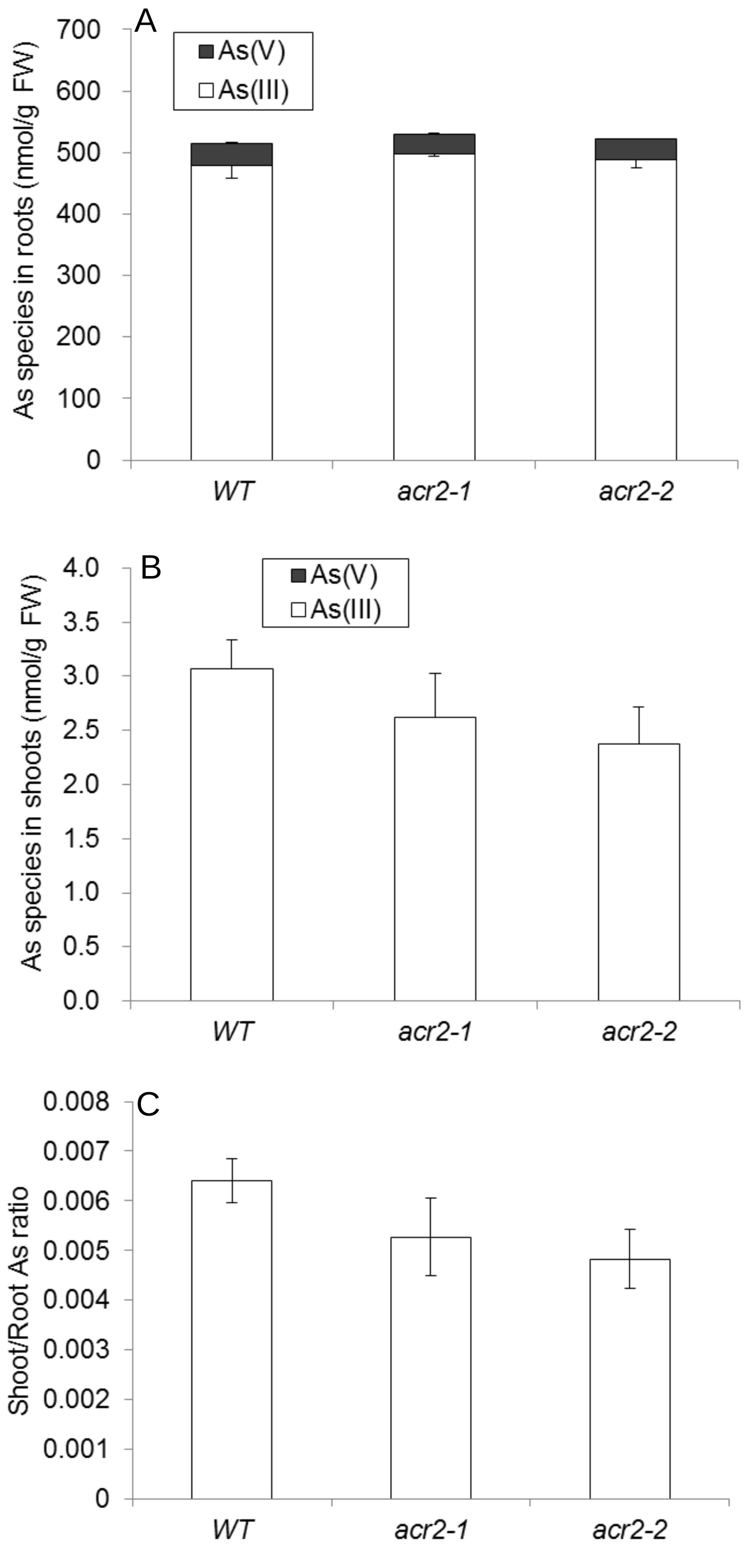

Figure S1. Arsenate (As(V)) reduction in *Arabidopsis thaliana* wild-type and *AtACR2* mutants. As speciation in roots (A), shoots (B) and the ratio of shoot to root As concentration (C). Plants were exposed to 25 µM As(V) in nutrient solution with 0.4 mM phosphate for 1 week .
